# The Avantgarde Carbostent in Patients Scheduled for Undelayable Noncardiac Surgery

**DOI:** 10.1155/2012/372371

**Published:** 2012-02-19

**Authors:** Carlo Briguori, Gabriella Visconti, Francesca De Micco, Amelia Focaccio

**Affiliations:** Laboratory of Interventional Cardiology, Department of Cardiology, Clinica Mediterranea, Via Orazio 2, 80121 Naples, Italy

## Abstract

*Background*. Treatment of patients who need coronary revascularization before undelayable non-cardiac surgery is challenging. *Methods*. We assessed the safety and efficacy of percutaneous coronary interventions (PCI) using the Avantgarde^ TM^ Carbostent (CID, Italy) in patients undergoing PCI before undelayable non-cardiac surgery. The Multiplate analyzer point-of-care was used to assess residual platelet reactivity. One major cardiac events (MACE, defined as death, myocardial infarction, and stent thrombosis and major bleeding) were assessed. *Results*. 42 consecutive patients were analyzed. Total stent length ≥25 mm was observed in 16 (37%) patients. Multivessel stenting was performed in 11 (31.5%) patients. Clopidogrel was interrupted 5 days before surgery in 35 patients, whereas it was stopped the day of the surgery in 7 patients. Surgery was performed after 27 ± 9 (7–42) days from PCI. MACE occurred in one patient (2.4%; 95% confidence interval: 0.01–13%), who had fatal acute myocardial infarction 3 days after abdominal aortic aneurysm surgery and 12 days after stent implantation. No case of major bleeding in the postoperative phase was observed. *Conclusions*. The present pilot study suggests that, although at least 10–14 days of dual antiplatelet therapy remain mandatory, the Avantgarde^ TM^ stent seems to have a role in patients requiring undelayable surgery.

## 1. Introduction

Decision-making for high cardiovascular risk patients requiring undelayable surgical procedure is still challenging. Undelayable surgery may be necessary in patients suffering from malignancy, high rupture risk aneurismal disease, acute abdominal disease, and pharmacological uncontrolled pain due to orthopedic or neurological disease. The current guidelines are very restrictive to the prophylactic myocardial revascularization before noncardiac surgery [1]. In this condition percutaneous coronary revascularization (PCI) should be preferred to coronary artery bypass surgery (CABG) [1]. Due to the need for dual antiplatelet therapy (DAPT), the current guidelines recommend to delay surgery until after the time window required for DAPT, that is 30 days for bare-metal stents (BMSs) and 1 year for drug-eluting stent [1]. However, the 30-day DAPT may represent an unacceptable delay for some patient scheduled for undeferrable noncardiac surgery. In this challenging scenario, the risk of major cardiac event, due the severe coronary artery disease, should be balanced to the risk of stent thrombosis, associated with premature DAPT discontinuation. The reported rate of stent thrombosis in this setting ranges from 4% to more than 50% [2–5]. Stent thrombosis seems to be caused by the premature discontinuation or reduction of DAPT, the thrombotic risk associated with many forms of major surgery, or an interaction between the two.

New stent types, allowing more rapid reendothelialization, may minimize the risk of stent thrombosis [6]. The Avantgarde Carbostent (CID, Saluggia, Italy) might represent a further solution. This new stent combines the unique characteristics of the Carbostent family (integral Carbofilm coating, close cell design) with a thinner and optimized stent strut, which has an impact on the rapidity of the endothelialization process, reducing the risk of thrombosis and safety issues.

 Therefore, we investigated the clinical performance, efficacy, and the complication rate of this new device in a pilot study of consecutive patients with indication for percutaneous coronary interventions before undeferrable noncardiac surgery.

## 2. Matherials and Methods

### 2.1. Patients Population

All consecutive patients requiring coronary revascularization before an undelayable major noncardiac surgery in our Institution from October 2009 to September 2011 were included in the present study. Patients were candidate for coronary revascularization before noncardiac surgery only if they fulfilled the following criteria: (a) patients with unstable angina or acute coronary syndromes, (b) stable patients with left main disease or three vessel disease or 2-vessel disease with proximal left anterior descending artery disease and either left ventricular ejection fraction <50% or inducible ischemia, and (c) stable patients non controlled with optimal medical therapy [7]. All patients were treated by PCI. The surgical procedures were categorized according to the surgical risk, based on the Revised Cardiac Risk Index [8]. The risk of noncardiac surgery was defined high and low. High risk included abdominal, vascular, thoracic, and head and neck; low risk included urologic, orthopedic, breast, and skin [9].

### 2.2. Percutaneous Coronary Intervention and Noncardiac Surgery

Patients received intracoronary isosorbide dinitrate (0.1–0.3-mg) prior to initial and final angiograms to achieve maximal vasodilatation Percutaneous coronary intervention was performed by implantation of a Avantgarde stent (CID, Saluggia, Italy) in all instances. Stents were implanted according to current clinical practice. Direct stenting was performed according to the operator's preference. All patients received aspirin 325 mg and clopidogrel (75 mg daily) before stent deployment, with a loading dose (600 mg of clopidogrel) given to patients not pretreated. All patients received unfractionated heparin (70 IU/Kg) or bivalurdin (0.75 mg/kg prior to the start of the intervention, followed by infusion of 1.75 mg/kg per hour or 1.0 mg/kg per hours in patients with chronic kidney disease for the duration of the procedure) in order to achieve and activated clotting time >250 seconds. Glycoprotein IIb/IIIa inhibitors were administered according to operator preference.

### 2.3. Point-of-Care Platelet Function Testing

**Figure 1:**
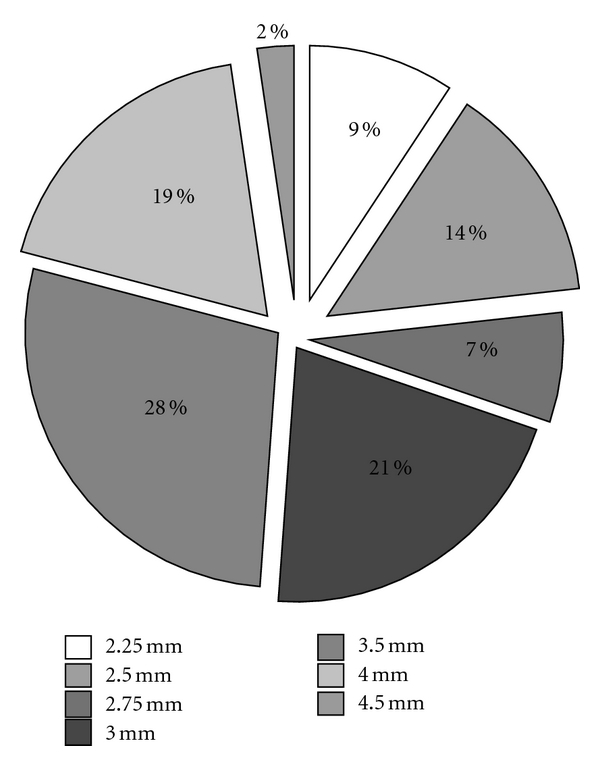
Platelet aggregation assessed by the point-of-care Multiplate analyzer (AU: arbitrary aggregation units (AUs) that are plotted against time: AU·min). According to the AU·min value, the following groups were identified: enhanced responders (≤188 AU·min), low responders (≥468 AU·min), and normal responders (189 to 467 AU·min). Time of clopidogrel interruption before surgery was defined according to the Au-min value.

The ADP-induced platelet aggregation in whole blood was assessed within 24 hours after stent implantation with multiple electrode platelet aggregometry (MEA) using a new-generation impedance aggregometer called Multiplate analyzer (Dynabyte) [10, 11]. After 1 : 2 dilution of whole blood with 0.9% NaCl solution and stirring for 3 min in the test cuvettes at 37°C, 6.4 *μ*mol/L ADP was added. Platelet aggregation was continuously recorded for 5 min. Impedance with MEA is transformed to arbitrary aggregation units (AUs) that are plotted against time (AU·min). According to the AU·min value, the following groups were identified: enhanced responders (≤188 AU·min), low responders (≥468 AU·min), and normal responders (189 to 467 AU·min) [10, 11]. Although not supported by previous studies, these values were used to guide the time of clopidogrel interruption. Indeed, in normal and high-responders patients, clopidogrel was withdrawn 5 days before surgery, due to the expected higher risk of bleeding than thrombosis. On the contrary, in the low-responders patients, clopidogrel was withdrawn the day of the intervention, due to the expected higher tisk of thrombosis than bleeding [11] (Figure 1). It was restarted with a loading dose of 600 mg as soon as possible after surgery, according to the clinicians discretion and patients' conditions [12]. The use of aspirin was decided by the surgeon.

### 2.4. Avantgarde Carbostent

The coronary system Avantgarde Carbostent consists of a stent made of L605 cobalt-chromium alloy (stellite 25) with a strut thickness of 70 *μ*m or 80 *μ*m (for diameter ≥3.0 mm) that is integrally covered with i-Carbofilm. This second generation extremely thin (≤0.3 *μ*m) coating is made of pure carbon atoms, which provides superior bio-haemocompatibility, resulting in stent thromboresistance [13].

### 2.5. Study Objective

The primary objective of the study was the assessment of the clinical performance of the Avantgarde Carbostent in patients undergoing undeferrable noncardiac surgery. The following in-hospital and 1-month major adverse cardiac events (MACEs) were analyzed: cardiac death, nonfatal myocardial infarction, stent thrombosis, and major bleeding. Myocardial infarction was defined as the association of ≥1 clinical and ≥1 biological criteria: acute-onset chest pain and/or typical modification on electrocardiogram (ST- or T-wave modification or new left bundle branch block) and an increase in troponin >99th percentile of the upper reference limit [14]. Stent thrombosis was classified according to the definition proposed by the Academic Research Consortium [14]. Major bleeding was defined as intracranial, intraocular, or retroperitoneal haemorrhage, clinically overt blood loss resulting in a decrease in haemoglobin of more than 3 g/dL, any decrease in haemoglobin of more than 4 g/dL, or transfusion of 2 or more units of packed red blood cells or whole blood [15].

### 2.6. Statistical Analysis

Continuous variables are presented as mean ± 1 standard deviation or as median and interquartile ranges when appropriate. Categorical variables were analyzed by Chi-square test.

## 3. Results

### 3.1. Patients Characteristics

Forty-two patients were included into the current study. The type of noncardiac surgery according to the risk is reported in Table 1. The clinical and angiographic characteristics are depicted in Tables 2 and 3. The indication for myocardial revascularization was class Ia in 35 (83%) patients and class IIa in 7 (17%) patients. Surgery was performed after  27 ± 9  (7–42) days from stent implantation. Duration of DAPT was  22 ± 8  (6–41) days. According to the AU·min value, 43% of patients were enhanced responders (≤188 AU·min), 16.5% were low responders (≥468 AU·min), and 40.5% normal responders (189 to 467 AU·min).

### 3.2. Procedural Characteristics

**Figure 2:**
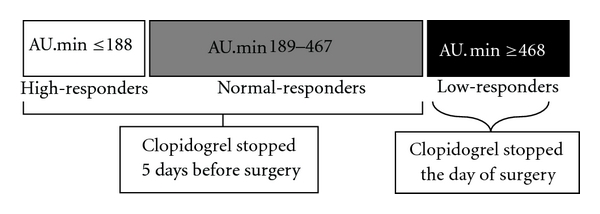
Distribution of the stent diameter in the global population.

Angiographic success was obtained in all patients. More than 1 stent was implanted in 8 patients (19%). Direct stenting was performed in 15 patients (21%). Rotational atherectomy was performed in one patient. Glycoprotein IIb/IIIa inhibitor was used in only 1 patient. Bivalirudin was used in 11 (25.6%) patients. Six (14%) patients had 2 or more stents. Distribution of stent diameter is represented in Figure 2. Total stent length ≥ 25 mm was observed in 16 (37%) patients. Multivessel stenting was performed in 11 (31.5%) patients. Periprocedural myocardial infarction occurred in 14 (32.5%) patients. The quantitative coronary angiography characteristics are reported in Table 4.

### 3.3. Clinical Outcome

According to the residual platelet reactivity, clopidogrel was stopped the day before the surgery in the 7 (16.5%) low-responders patients. In all the others, both aspirin and clopidogrel administration were interrupted 5 days before the scheduled surgery. There was only one case (2.4%; 95% confidence interval: 0.01–13%) of MACE at 1 month. The patient had fatal acute anterior myocardial infarction 3 days after abdominal aortic aneurysm surgery and 12 days after stent implantation (Avantgarde 3.0 × 25 mm) in the proximal left anterior descending artery. This patient was affected by chronic kidney disease and had periprocedural myocardial infarction: due to the high residual platelet reactivity, clopidogrel was discontinued the day before surgery. No case of major bleeding in the postoperative phase was observed.

## 4. Discussion

The current pilot study suggests that (1) the Avantgarde Carbostent may represent a promising new stent to be selected in PCI performed before undeferrable noncardiac surgery, and (2) the Multiplate analyzer point-of-care platelet function testing may be used to guide the time of clopidogrel administration interruption.

Kaluza et al. firstly reported that patients undergoing noncardiac surgery within 6 weeks of BMS implantation have a 20% death rate; the risk is almost limited to those patients treated in the first 2 weeks [2]. The majority of deaths were caused by acute myocardial infarction and stent thrombosis. Other experiences reported a MACE rate ranging from 3.9% to 86% [3–5, 16–18]. Noncardiac surgery after stent implantation remains a delicate balance between the deleterious stent thrombosis with myocardial infarction, the potential risk of increased intra- and postoperative blood loss, hypercoagulability attributable to the surgical trauma, and the perioperative stress [2, 3, 16, 19]. Noncardiac surgery increases the risk of stent thrombosis, especially when the procedure is performed early after stent implantation, likely because stents are not yet endothelialized, because DAPT is often discontinued in the periprocedural period and because surgery creates a prothrombotic state [20, 21]. The stress response to major surgery includes sympathetic activation promoting shear stress on arterial plaques, enhanced vascular reactivity inducing vasospasm, reduced fibrinolytic activity, platelet activation, and hypercoagulability. This concern highlighted the importance of (1) limit revascularization only to high-risk patients [22–24] and (2) delaying surgery when possible and continuing DAPT in the perioperative period when surgery is not delayed [12]. When revascularization is required, PCI with eventual BMS is recommended [1].

### 4.1. Time of Clopidogrel Discontinuation

There is a general agreement that premature DAPT discontinuation represents the major cause of in-stent thrombosis, leading to myocardial infarction or even death. Indeed the MACE rate was very low in patients operated on who continued DAPT [5, 17]. On the other hand, the incidence of bleeding is high when DAPT is continued [25]. The ACC/AHA guidelines recommend to delay surgery until after the time window required for DAPT, 30-day for BMS and 1 year for DES [1]. However, the 30 days DAPT may represent an unacceptable delay for some patient scheduled for undeferrable noncardiac surgery. In the present pilot study, we observed that the Multiplate analyzer point-of-care platelet function testing may be used to guide the time of clopidogrel administration interruption. Indeed, in low-responders patients clopidogrel might be stopped the day of the surgery. An alternative strategy has been proposed by Savonitto et al. in 30 patients with a recently implanted drug eluting stent (1–12 months) and undergoing undeferrable noncardiac surgery. This approach, named “bridging strategy”, includes tirofiban (an intravenous short-acting glycoprotein IIb/IIIa receptor blocker) in the perioperative period in order to temporary replace oral clopidogrel, interrupted 5 days before the intervention [26]. No adverse cardiac events during the index hospitalization were observed. On the contrary, there was 1 case of major bleeding and 5 cases of minor bleedings. Finally, ticagrelor, due to its short half-life and the observed less surgery-related bleeding events, may represent a valuable clinical alternative in this subset of patients [27]. Ticagrelor, indeed, is the first reversibly binding oral P2Y12 receptor antagonist that blocks ADP-induced platelet aggregation and exhibits rapid onset and offset of effect, which closely follow drug exposure levels.

### 4.2. Stent Platform

New stent types, allowing more rapid re-endothelialization, may minimize the risk of stent thrombosis and therefore may represent an alternative strategy in this high-risk population. The Avantgarde Cardofilm has some potentially favorable characteristics, including (1) a reduced strut thickness (70–80 *μ*), (2) the Co-Cr alloy, and (3) the i-Carbofilm coating. Haemocompatibility is one the main requisites of materials used in the making of coronary stents. As compared to the endothelium, the natural vascular surface, all the artificial surfaces activate platelets and coagulation plasmatic factors. In order to improve blood compatibility, surface treatments are performed on biomaterials. One of these treatments consists of coating the material made of pyrolytic carbon. This is an artificial material made of carbon microcrystals with a high-density turbostratic structure. It has been demonstrated that this Carbofilm coating shows a lower thrombogenicity than the uncoated material, as it induces minimal platelet adhesion and limits the activation of the intrinsic pathway of coagulation [28]. *In vitro *studies have shown a quick growth and attachment of endothelial cells in the Carbofilm-coated samples [29]. In an *in vivo *optical coherence tomography study enrolling 20 patients with acute myocardial infarction treated by the Avantgarde Carbostent implantation, Prati et al. reported that observed a quite complete stent struts coverage after only 3–7 days. The stent level analysis, indeed, showed an overall percentage of uncovered struts of 4.4%  ±  3.4% (range 0.3–12.1%) [30]. The coronary stents belonging to the Carbostent family have been already tested in 3 studies [10–12], designed specifically to evaluate the safety profile of these devices in patients receiving aspirin alone (ANTARES and SIMPLE) or comparing the safety performances in patients receiving aspirin and thienopyridine versus aspirin alone (SAFE). The results of these 3 clinical studies demonstrated the excellent safety profile of the Carbostent devices.

Antibody-coated stents that can attract endothelial progenitor cells have been also proposed. Piscione et al. recently reported a pilot study of 30 patients treated by the Genous “prohealing” stent, followed by a short-term DAPT, before undeferrable urgent of live-saving noncardiac surgery [6]. The Genous bioengineered R stent (OrbusNeich medical Technologies, Fort Lauderdale, Fla, USA) is coated by anti-hCD34. This stent should lead to a rapid vessel and strut healing, due to its properties of capturing circulating CD-34+, progenitor cells to the luminal stent struts [31]. Antiplatelet therapy was stopped after 12.2 ± 3.9 days after stent implantation. Noncardiac surgery was performed 10 to 22 days after stenting (average 17.2 ± 3.9 days). Undelayable surgical procedures were high risk in 67% of patients. No patients suffered from MACE or bleeding in the perioperative period.

### 4.3. Study Limitations

The small sample size and the lack of a control group represent the major limitations of the present registry. Indeed, this is a pilot study designed to test the hypothesis of the advantages of Avantgarde Carbostent implantation in this high-risk population. Furthermore, the safety of clopidogrel interruption guided by the Multiplate analyzer point-of-care platelet function testing should be tested in a larger population.

## 5. Conclusions

The present pilot study suggests that the Avantgarde Carbostent may represent a valuable alternative to treat patients with severe coronary artery disease and undelayable noncardiac surgery. Further studies are needed to confirm this result in a larger population.

## Figures and Tables

**Table 1:** Type of noncardiac surgery according to the risk*.

	Number of patients (*n* = 42)
High-risk surgery	40
Abdominal	10
Vascular	23
Thoracic	7
Low-risk surgery	2
Urologic	1
Orthopedic	1
Revised cardiac risk index^#^	
1 risk factor	2 (5%)
2 risk factors	33 (76.5%)
3 or more risk factors	8 (18.5%)

The risk was defined according to Eagle et al. [9]. The revised cardiac risk index was according to Lee et al. [8].

**Table 2:** Clinical characteristics of the enrolled patients.

	Patients (*n* = 42)
Age, yrs (mean ± SD)	73 ± 9
Male (%)	33 (79%)
BMI (kg/m^2^)	26 ± 3
Symptoms	
Stable angina	29 (69%)
Unstable angina	13 (31%)
Diabetes mellitus	13 (31%)
Chronic kidney disease	18 (42%)
Hypertension (%)	32 (76%)
Current smoker (%)	9 (21.5%)
Prior MI (%)	14 (33%)
Prior PCI (%)	4 (9.5%)
Prior CABG (%)	6 (16%)
LVEF, % (mean ± SD)	49 ± 9

**Table 3:** Angiographic characteristics of the enrolled patients.

	Total population (*n* = 42)
Distribution of coronary artery disease	45
1-vessel	13 (29%)
2-vessel	13 (29%)
3-vessel	19 (42%)
Vessel treated	45
Left main	5 (11%)
LAD	23 (51%)
LCX	14 (31%)
RCA	3 (7%)
Lesion site	49
ostial	8 (16%)
proximal	20 (41%)
midvessel	17 (35%)
distal	4 (8%)
Lesion type	49
A	7 (14.5%)
B1	16 (32%)
B2	19 (39%)
C	7 (14.5%)
Bifurcation lesions	13 (30%)
Double stenting technique	5 (11.5%)

Chronic total occlusion	2 (4.5%)

Number of treated vessel/patient	1.1 ± 0.5

Number of treated lesion/patient	1.2 ± 0.7

LAD: left anterior descending artery; LCX: left circumflex artery; RCA: right coronary artery.

**Table 4:** Quantitative coronary angiography and procedural characteristics of the enrolled patients.

	Total population (*n* = 42)
Diameter stenosis, %	
Pre	81 ± 9
Post	2 ± 2
Reference vessel diameter, mm	
Pre	3.2 ± 0.6
Post	3.4 ± 0.6
Minimal lumen diameter, mm	
Pre	0.53 ± 0.43
Post	3.3 ± 0.5
Lesion length, mm	16 ± 5
Maximum balloon-to-artery ratio	1.19 ± 0.22
Postdilatation (semicompliant balloon)	45 (83%)
Final pressure (atmospheres)	20 ± 5
Total stent length/patient, mm	24 ± 11
